# A Sawn Timber Tree Species Recognition Method Based on AM-SPPResNet

**DOI:** 10.3390/s21113699

**Published:** 2021-05-26

**Authors:** Fenglong Ding, Ying Liu, Zilong Zhuang, Zhengguang Wang

**Affiliations:** College of Mechanical and Electrical Engineering, Nanjing Forestry University, Nanjing 210037, China; dfl@njfu.edu.cn (F.D.); zzl0702@njfu.edu.cn (Z.Z.); nanlinwzg@njfu.edu.cn (Z.W.)

**Keywords:** recognition of sawn timber, deep learning, attention mechanism, spatial pyramid pooling

## Abstract

Sawn timber is an important component material in furniture manufacturing, decoration, construction and other industries. The mechanical properties, surface colors, textures, use and other properties of sawn timber possesed by different tree species are different. In order to meet the needs of reasonable timber use and product quality of sawn timber products, sawn timber must be identified according to tree species to ensure the best use of materials. In this study, an optimized convolution neural network was proposed to process sawn timber image data to identify the tree species of the sawn timber. The spatial pyramid pooling and attention mechanism were used to improve the convolution layer of ResNet101 to extract the feature vector of sawn timber images. The optimized ResNet (simply called “AM-SPPResNet”) was used to identify the sawn timber image, and the basic recognition model was obtained. Then, the weight parameters of the feature extraction layer of the basic model were frozen, the full connection layer was removed, and using support vector machine (SVM) and XGBoost classifier which were commonly used in machine learning to train and learn the 21 × 1024 dimension feature vectors extracted by feature extraction layer. Through a number of comparative experiments, it is found that the prediction model using linear function as the kernel function of support vector machine learning the feature vectors extracted from the improved convolution layer performed best, and the F1 score and overall accuracy of all kinds of samples were above 99%. Compared with the traditional methods, the accuracy was improved by up to 12%.

## 1. Introduction

Sawn timber refers to a type of solid wood board whose size meets the industry standard, and its specifications are unified after a series of processing procedures, such as peeling, cutting, and polishing. Compared with logs, sawn timber has the advantages of small deformation, crack resistance, high bonding strength, diverse colors, and easy splicing. It is a common green and sustainable multifunctional material used in the furniture, decoration, and construction industries. The properties of sawn timber from different tree species differ in color depth, density, softness, bending strength and tensile strength, so each kind of sawn timber has different uses [1,2]. Owing to the decrease and shortage of log resources in the world, many timber producing and exporting countries have begun to prohibit or restrict log exports [3]. However, the human requirements for the appearance, bearing capacity, quality, and other attributes of wood products are gradually increasing. In order to meet the high-quality demands of human beings for timber products under the condition of increasingly tense shortage of log resources, meet the needs of reasonable use of timber products and at the same time ensure the quality of products, it is necessary to identify the sawn timber according to tree species, so as to ensure the best use of materials.

Traditional sawn timber identification mainly depends on the subjective naked eye judgment of experienced workers. The accuracy of these judgements is low because it is easy to miss features and defects and misjudge quality. However, the emergence of machine vision and machine learning has gradually begun to automate this task. Using a machine learning strategy to analyze the image data of sawn timber will accelerate the production efficiency and improve the quality of sawn timber manufacturing. The general process of machine learning includes data acquisition, characteristic engineering, and mathematical modeling. Machine vision technology is a common data acquisition method in the fields of object recognition and identification. Common data acquisition tools include RGB cameras [4], spectral cameras [5,6,7], lasers [8], and other optical instruments.

In the process of establishing features, the commonly used feature extraction methods for wood materials include the gray-level co-occurrence matrix [9,10,11], local binary pattern (LBP) [12,13,14], scale-invariant feature transform [15,16], and wavelet transform [17,18]. In addition, modified versions or other methods based on the above methods have emerged. Xie [19] proposed a wood surface defect detection method based on Tamura and GLCM mixed features and used a BP neural network to identify wood images; the highest recognition rate was 90.67%, which effectively guaranteed the accuracy and robustness of the algorithm. Barmpoutis [20] proposed a new spatial descriptor that regards each image as a set of multidimensional signals. More specifically, this method can represent the timber image as a series histograms of high-order linear dynamic systems generated by vertical and horizontal image blocks; it then uses a support vector machine (SVM) classifier to identify timber cross-sectional images, with an identification accuracy of 91.47%. Sugiarto [21] extracted the gradient histogram of wood images and used an SVM to identify the wood; the accuracy was only 77.5%. Although the above feature extraction methods can extract the image features of the sawn timber surface to some extent, the extracted features often have limited recognition ability for strange samples with poor generalization and robustness. From the above work, it can be found that artificial neural networks and SVMs are commonly used algorithms in wood identification.

Deep learning is a new research direction in the field of machine learning. Convolutional neural networks (CNNs) are typical examples of deep learning. A CNN obtains the feature map of the surface image of the object to be measured through the convolution layers connected before and after the former, and then converts it into a one-dimensional vector and puts it into the full connection layer, where a linear regression function is used for identification. With its excellent image feature extraction ability and representation learning ability, it is widely used in many fields that need machine vision, such as medical inspection [22], food detection [23], forest protection [24], remote sensing [25], fault diagnosis [26] and so on. However, when using a CNN for image identification, the image must be cut or resized to a fixed square size, such as 224 × 224 or 300 × 300, and then the resized images can be used for feature extraction and input into the full connection layer for identification. However, there are few studies on the identification of standardized sawn timber produced by mechanical processing, which is mostly rectangular with a variety of specifications. Cutting or stretching processing will lose or distort a large amount of image information [27].

In order to solve the problem of image information loss and improve the accuracy of sawn timber tree species classification, this paper makes the following attempts: (1) Combining the classical machine learning algorithms with the deep learning strategies, establishing the relationship between the characterization images and the tree species of sawn timber. (2) Using the attention mechanism [28,29,30] and spatial pyramid pooling strategy to modify the convolution layer of ResNet network (simply called “AM-SPPResNet”), so as to eliminate the limitation of image input size in convolutional neural network, and improve the feature extraction ability of sawn timber characterization images significantly. (3) Using support vector machine and XGBoost classifier instead of linear classifier of full connection layer to learn and identify the image features extracted by the improved convolution layer. The contribution of this paper is as follows: using machine learning strategies to establish a new relationship between sawn timber characterization images and sawn timber species, promoting the realization of automatic identification of sawn timber species, so as to give full play to the maximum use value and economic value of each piece of sawn timber, accelerate the work efficiency of sawn timber manufacturing industry, increase economic benefits, and reduce the waste of natural resources.

## 2. Materials and Methods

### 2.1. Acquisition and Partition of Dataset

**Figure 1:**
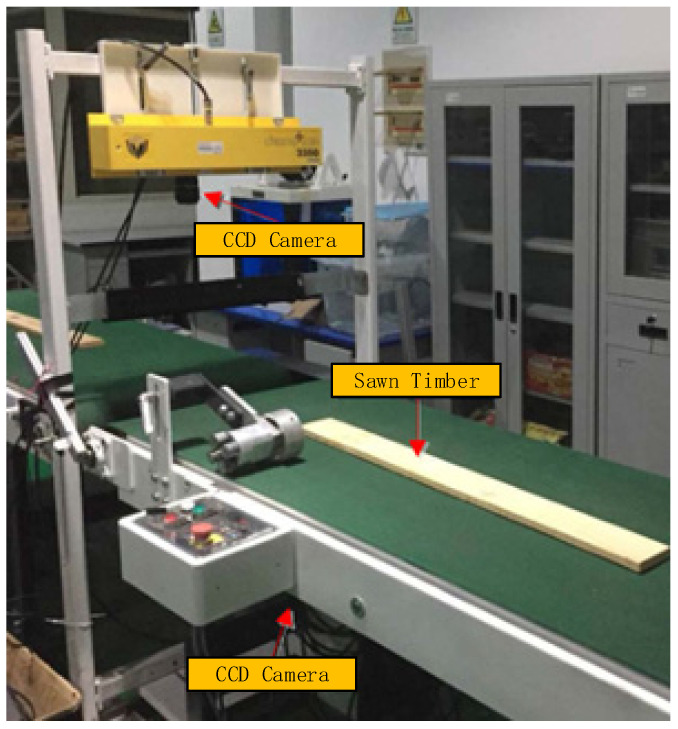
Image acquisition equipment for sawn timber.

A sawn timber surface image acquisition equipment system was specially built for this study, as shown in Figure 1. The image acquisition equipment is mainly composed of two conveyor belts, two linear CCD cameras, a light source and an infrared sensor. The distance between the two belts is 15 cm. When the sawn timber moved forward on the conveyor belts, the linear scan cameras distributed on both sides of the conveyor belts were triggered by the infrared photoelectric sensor installed on the side to collect the double-sided image of the sawn timber in the space between the two conveyor belts. Surface images of sawn timber made of beech wood, ash wood, birch wood, cherry wood, and fir wood were obtained using this equipment. The software, hardware, and environment configurations are shown in Table 1.

**Figure 2:**
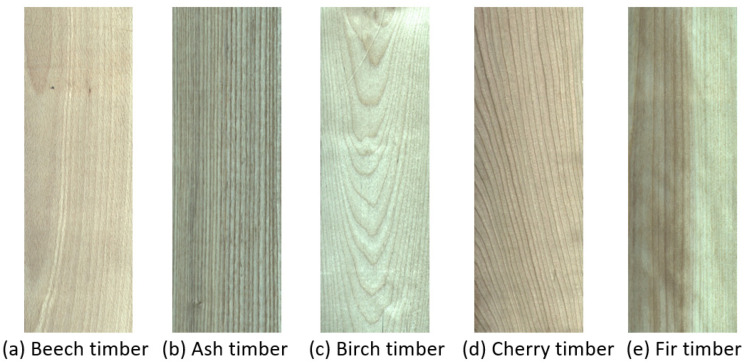
Color images of sawn timber.

Sawn timber can have any size. Therefore, to minimize the information loss in the process of image deformation, this study decided to simply scale the original image to 1/5 of the original size in equal proportion, without using image clipping or scaling to a unified specification. Final color images of the sawn timber surfaces are shown in Figure 2.

Due to the small dataset, in order to obtain a stable and efficient identification model of sawn timber, this paper decided to use K-fold cross validation method instead of the traditional hold-out method to divide the dataset. The traditional hold-out method simply divides the data set into three groups: training set, validation set and test set. It is very sensitive to the proportion of samples used in the division, whether the distribution of various types of data in each group is the same as that of the original data set, and the optimal models obtained by different divisions are often different. After being divided into three groups, fewer data are used for training, which is more unfavorable to the small-scale data set used in this paper. 

The advantage of K-fold cross-validation method is that it can ensure each sample is involved in training and testing, reduce the impact of insufficient training caused by small data set, and can significantly reduce the generalization error of the model. But k is not the greater the better, k is too large means too many times of training, large amount of calculation. And the larger the k, the smaller the sample size of each set of test sets, which cannot well reflect the generalization performance of the model. However, in principle, the test set should not be involved in the process of model training and parameter adjustment. Therefore, the original data set was divided into training set and test set at a ratio of 8:2, and then the training set was trained by K-fold cross-validation method, with k of 4, that is, 4-fold cross-validation. The distribution of tree species in each set of data is shown in Table 2.

### 2.2. Convolutional Neural Networks

Convolutional neural networks (CNNs) are a kind of feedforward neural network with convolution operation and deep structure. CNNs generally composed of an input layer, convolution layer, down sampling layer (also known as pooling layer), full connection layer, and output layer. CNNs not only have the advantages of good fault tolerance, self-adaptability, and strong self-learning ability of traditional neural networks, but also have the advantages of automatic feature extraction, weight sharing, and a good combination of input images and network structure [31]. With its excellent image feature extraction ability and representation learning ability, it is widely used in many fields that need machine vision, such as drug detection, medical examination, fault diagnosis and so on.

**Figure 3:**
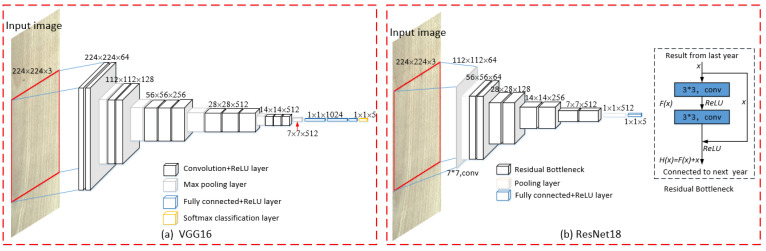
VGG and ResNet network structure diagram.

As convolutional neural networks such as AlexNet [32], VGGNet [33], ResNet [34] and Inception [35] have developed to a deeper level, and at the same time, they have also developed in the direction of the width and the fusion of the front and rear convolution layers. They have constantly updated the highest record of the competition in the field of image recognition, and the image recognition has made more accurate and rapid progress. VGGNet has smaller convolution kernels and deeper layers than AlexNet, and has good generalization performance, but the biggest problem is too many parameters; ResNet was proposed by Kaiming, He in 2015, through the use of Residual Unit, the 152-layer convolutional neural network was successfully trained, which mainly solved the degradation problem in the deep network, and the number of parameters was much smaller than that of VGGNet. The network structure of VGGNet and ResNet is shown in Figure 3:

It can be found from Figure 3 that the structures of both are similar, and they are stacked in the depth direction of convolution. However, ResNet introduces the residual bottleneck structure of jump connection in Figure 3a to realize the addition of upper features and lower features after convolution, which form a part of the output layer. The differential amplification of the training gradient is realized by the constant mapping relationship $\;H\left(x\right)=F\left(x\right)+x$ in the jump connection structure, so as to effectively avoid the problem of gradient disappearance caused by excessive depth in VGGNet network. When using deep network structure such as Resnet101, the feature extraction ability of the model is further improved, and the performance of the model is also improved.

In Figure 3, whether VGGNet or ResNet, there will be a full connection layer which is very similar to that of the artificial neural network, is composed of linear connection layers with different numbers of neurons, playing the role of “classifier”, and mapping the final results obtained from each layer before the full connection layer to the target category interval. A convolution operation itself has no size limitation on the size of the images, and it can generate feature maps of any size. However, the calculation of the full connection layer is equivalent to the inner product of the input feature map data matrix and the weight matrix of the full connection layer. When configuring the network, the parameter dimension of the full connection layer is fixed. In order to make the inner product of the two matrices, the dimension of the input feature map data matrix must also be fixed. This requires convolution neural network input image size must be fixed, such as 224 × 224, 300 × 300, etc. In order to obtain the required size of the model, it is often necessary to cut or stretch the sawn timber image of any size, but the cutting transformation will lead to the loss of image information, and the stretching transformation will lead to the distortion of image information, which will affect the accuracy of image recognition.

### 2.3. Optimized Convolutional Neural Network

In order to solve the above problems and improve the identification accuracy of sawn timber, this paper used visual attention mechanism and spatial pyramid pooling strategy to improve the convolution layer of ResNet101 network, and used SVM and XGBoost classifier to replace the linear discriminant classifier of full connection layer to train and learn the features obtained from convolution layer. 

**Figure 4:**
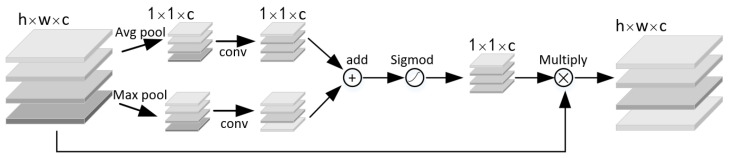
Schematic of the channel attention module.

The visual attention mechanism is a brain signal processing mechanism unique to human vision. By quickly scanning the global image, human can obtain the target area that needs to be focused on, and then invest more attention resources in this area to produce a more discriminative feature representation, while suppressing irrelevant information in other areas. This mechanism can effectively improve the efficiency of image recognition and cognition. In this paper, the attention mechanism of the sawn timber tree species recognition model was realized by constructing channel attention module and spatial attention module in residual network. The channel attention module (Figure 4) firstly performs average pooling and maximum pooling operations on the input feature map at the same time. The feature information obtained by average pooling operation mainly describes the background (i.e., irrelevant region) information of the image, and the feature information obtained by maximum pooling operation mainly describes the texture information of the image. At the same time, two pooling operations are used to more effectively describe the information contained in the feature channel. Two groups of pooled results are extracted by 1 × 1 convolution kernel and added to form a new feature map. Sigmod function is used to constrain the new feature map to (0,1) interval to enhance the expression of important information and suppress the expression of useless information.

**Figure 5:**
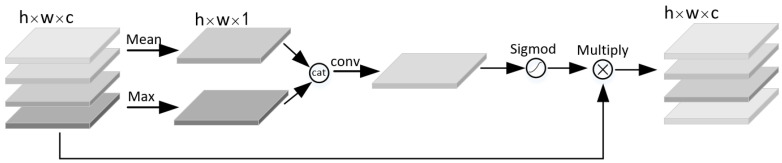
Schematic diagram of spatial attention module.

The spatial attention module (Figure 5) first calculates the mean and maximum values of each channel on the channel dimension of the input feature map to reduce the increase in the number of parameters. 

After the two groups of results are spliced, the information is extracted by convolution operation, and the mask map that describes the spatial position information of the feature map is obtained. After the mask is constrained and enhanced by Sigmod function, a new feature map describing the spatial position information of the sawn timber image is obtained, so as to enhance the expression of key information in the sawn timber image and suppress the expression of useless information.

**Figure 6:**
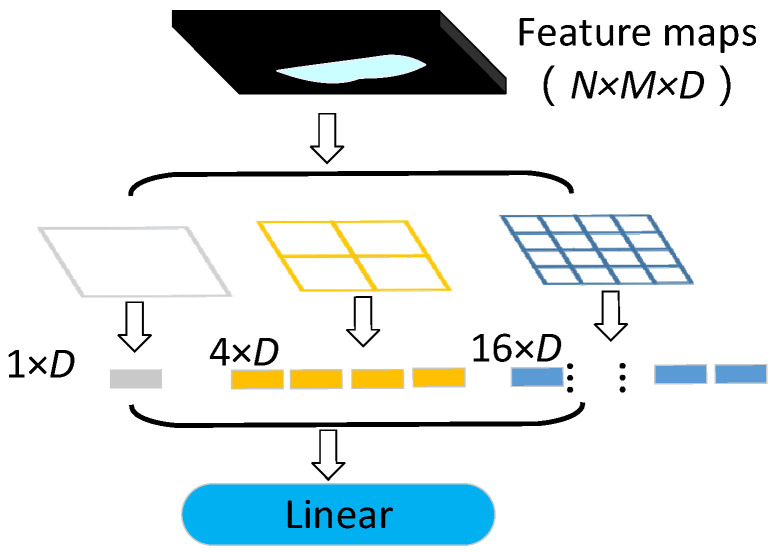
Schematic diagram of spatial pyramid pooling strategy.

The principle of spatial pyramid pooling strategy [27] is shown in Figure 6, the size of the feature map formed by the convolution operation of multi-layer convolution layer the multi-layer convolution layer is N × M × 1024, where the values of N and M depend on the size of the input image and the structure of the convolution layer. By using the mask of 4 × 4, 2 × 2, 1 × 1 to extract the feature map, we can obtain 16 + 4 + 1 = 21 different blocks. One feature is extracted from these 21 blocks, and the 21 × 1024 dimensional feature vectors can be extracted exactly. Whatever the size of the input image, the dimension of the input feature vectors obtained by the final classifier is fixed, which breaks the limit of the convolution neural network on the size of the input image.

**Figure 7:**
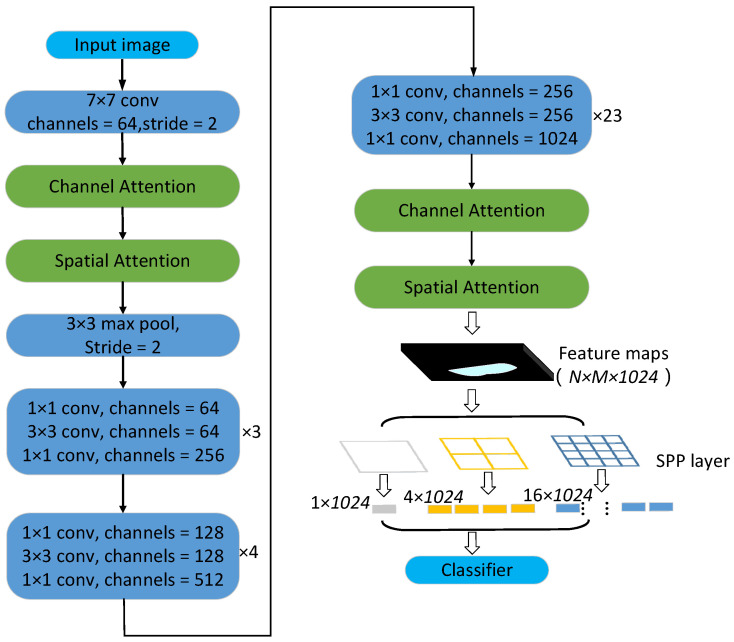
AM-SPPResNet Structure Graph for Feature Extraction.

The improved ResNet convolution network simply called AM-SPPResNet. Figure 7 shows the network structure used in this paper.

## 3. Results

### 3.1. Model Evaluation Index

In this paper, confusion matrix was introduced to evaluate the performance of the model trained by various methods, and the difference of identification performance of different methods in sawn timber data is compared. The confusion matrix can clearly show the identification results of the model on the test set [36]. Through the confusion matrix, four indicators representing the identification performance of the model, namely, accuracy rate, recall rate, accuracy rate and F1-Score, can be obtained. Their calculation methods are as follows:

(1) $$Precision=\frac{TP}{TP+FP}\;$$

(2) $$Recall=\frac{TP}{TP+FN}\;$$

(3) $$\mathrm{Accuracy}=\frac{TP+TN}{TP+TN+FP+FN}$$

(4) $$F1\;Score=\frac{2\times Precision\times Recall}{Precision+Recall}\;$$

where true positive (*TP*), false negative (*FN*), false positive (*FP*), true negative (*TN*), *Precision*, *Recall* and *F1-Score* are used to evaluate the predictive performance of the model for a specified class of samples in the test set, while accuracy is the prediction performance index of the evaluation model for all test samples. *TP* refers to the number of correctly predicted samples in the specified class samples, *FN* refers to the number of wrongly predicted samples in the specified class samples, *FP* refers to the number of wrongly predicted samples in other class samples, and *TN* refers to the number of samples in other class samples that are not predicted as the specified class. It can be concluded from the above formula that *Precision* rate refers to the proportion of samples predicted correctly in a given class sample. *Recall* rate refers to the proportion of correct samples in the samples predicted as a specified category. *Accuracy* refers to the proportion of all correctly judged samples in the total test samples. *F1 Score* is the harmonic mean between the precision rate and the recall rate, which is used to comprehensively reflect the overall performance index of the model for the specified category data. The values of the above evaluation indexes range from 0 to 1. The closer to 1, the better the classification performance of the model is, and vice versa.

### 3.2. Experimental Results

This paper first used a linear classifier as the full connection layer of the network, and used the learning rate of 1e−4 and batch-size of 16 and Adam optimizer to train the different convolutional neural network models. Then the weight parameters of the feature extraction layer of the model were frozen, and the feature extraction layer was used to extract the image feature vectors of the sawn timber. The dimension is 21 × 1024, which was used to train SVM (kernel = “linear”) and XGBoost (depth = 3, lr = 0.2) classifier respectively. After 4-fold cross validation, the identification performance shown in Table 3 was obtained.

In Table 3, the prediction matrix of the original resnet101 network and a series of improved versions in this paper are listed. It can be found that the identification performance of AM-SPPResNet, which introduces attention mechanism and spatial pyramid pooling strategy, has been greatly improved compared with the original network, and the accuracy rate has been improved by 5.3%, the range of F1 score value between different types was only 0.015, while the range of F1 score value of the original network was 0.108, it shows that the balance of AM-SPPResNet identification performance has also been greatly improved.

Using XGBoost and SVM instead of full connection layer to identify sawn timber tree species, the identification accuracy is increased by 0.5% and 2.5% respectively, indicating that this scheme was also beneficial to network classification. When the two schemes were combined, the identification accuracy of saw timber tree species was very close to 1. When AM-SPPResNet was combined with SVM, the precision, recall, F1 score and accuracy of various samples were all above 0.995. All the values on the prediction matrix were the average values of the results obtained by the four fold cross validation of various models, and the indexes on the prediction matrix obtained by our method in each fold training were all above 0.995, which indicates that the model has strong recognition ability for unfamiliar samples and is not easy to be disturbed by abnormal samples, that is, it has strong generalization and robustness. Table 4 shows the prediction matrix obtained by this method on the test set, indicating that it also has strong identification ability for unfamiliar samples.

## 4. Discussion

**Figure 8:**
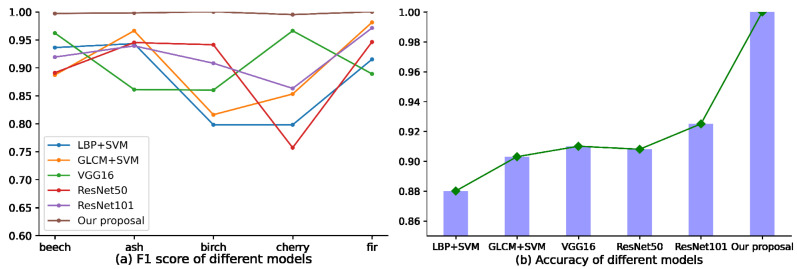
F1 score and Accuracy of different models in the identification of sawn timber species.

In this paper, the proposed identification model of sawn tree species was compared with traditional image feature extraction methods such as LBP and GLCM, and commonly used deep learning image identification methods such as VGG16 and ResNet. Results (Table 5 and Figure 8) show that the proposed method has obvious identification advantages. The identification accuracy obtained by the proposed method in this paper is up to 12% higher than that by the traditional image feature extraction method, and at least 7% higher than that by other convolutional neural network methods. 

However, the most important is the balance of performance evaluation between different models. It can be concluded from Figure 8a that the balance of the method proposed in this paper is also obviously advanced. The F1 score obtained by the proposed method in this paper were very close to each other on various samples, and the curves are very flat, but the other curves fluctuate greatly, indicating that the F1 scores obtained by them on various samples are quite different, especially in some categories. For example, traditional image feature extraction methods, such as LBP and GLCM, have lower precision rate, recall rate and F1 score than other samples in birch timber and cherry timber. It can be seen from the comparative experiments that the improvement of ResNet101 by using attention mechanism, spatial pyramid pooling strategy and SVM classifier has achieved the expected effect. The spatial pyramid pooling strategy eliminates the limitation of the network on the input size of the image and avoids the loss of image information. The attention mechanism focuses the network on useful feature information and inhibits the expression of useless features. The use of support vector machine instead of linear discriminant in the full connection layer can also further improve the identification accuracy. This should be related to the decision function of SVM. We all know that the classification layer of convolutional neural network is composed of multi-layer linear discriminator, which is similar to artificial neural network. Only when the amount of data is large and the computational power is strong can it show its advantages. When the data is insufficient, the performance of the classifier is not very good. SVM is a small sample learning method with solid theoretical foundation. Its final decision function is only determined by a small number of support vectors. The computational complexity depends on the number of support vectors rather than the dimension of the sample space. It can eliminate a large number of redundant samples while avoiding the dimension disaster. When the amount of data is small, the convergence speed is very fast.

In order to verify the universal applicability of our scheme, we conducted another comparative experiment on the “Wood-AUTH” dataset. The “Wood-AUTH” dataset [20] contains samples of normal wood structure of 12 kinds of wood (Table 6, three kinds of softwood and nine kinds of hardwood) existing in Greece. The results of comparative experiments are shown in Table 7.

It can be concluded from Table 7 that the network model proposed in this paper still obtains good classification results on the “Wood AUTH” dataset, and the prediction time of a single image is shorter than that in Table 5, which may be due to the smaller image pixels in the “Wood AUTH” dataset.

## 5. Conclusions

Efficient identification of sawn timber species is conducive to promoting the rational classification and use of sawn timber, maximizing the value of sawn timber and reducing wood resources waste. In this paper, the attention mechanism and spatial pyramid convolution strategy were used to improve the ResNet101 network, so that the image information was completely input into the model and the effective focus area was obtained. The support vector machine was used to replace the linear classifier to identify the features extracted by the convolution layer. Finally, the identification accuracy of sawn timber was improved to more than 99%, and the identification performance between various types was more balanced.

The method described in this paper can effectively solve the identification problem of sawn timber, but the parameters are too many, and the convolution neural network has a large amount of calculation, long operation time and poor real-time performance, which cannot be applied to the factory manufacturing end. It is necessary to explore a lighter and lighter deep learning feature extraction model, which can ensure the high performance of identification, shorten the operation time, improve the real-time performance of recognition, effectively meet the needs of the sawn timber manufacturing industry, and accelerate the development of the industry to intelligent manufacturing.

## Figures and Tables

**Table 1:** Software and Hardware Environment Configurations.

	Parameter
Cameras	DALSA LA-GC-02K05B
Camera lens	Nikon AF Nikkor 50 mm f/1.8 D
System	Windows 10 × 64
CPU	Intel Xeon W-2155@3.30 GHz
GPU	Nvidia GeForce GTX 1080 Ti (11G)
Environment configuration	Pytorch1.7.1 + Python3.7.0 + Cuda10.1

**Table 2:** Distribution of sawn timber sample data using 4-fold cross validation method.

Dataset		Beech Timber	Ash Timber	Birch Timber	Cherry Timber	Fir Timber
Train dataset	Fold 1	161	159	161	160	193
	Fold 2	161	159	161	160	194
	Fold 3	161	159	161	160	194
	Fold 4	162	160	161	160	194
Test dataset		162	160	161	161	194
total		807	797	805	801	969

**Table 3:** Identification performance of SVM and XGBoost on AM-SPPResNet feature.

	**ResNet101**					**AM-SPPResNet**				
	**Beech**	**Ash**	**Birch**	**Cherry**	**Fir**	**Beech**	**Ash**	**Birch**	**Cherry**	**Fir**
Precision	0.895	0.900	0.970	0.970	0.990	0.943	0.985	1.000	0.998	0.983
Recall	0.970	0.995	0.875	0.833	0.953	1.000	0.983	0.958	0.968	0.970
F1-Score	0.919	0.939	0.908	0.863	0.971	0.970	0.985	0.978	0.982	0.976
Accuracy	0.925					0.978				
	**ResNet101 + XGBoost**					**ResNet101 + SVM**				
	**Beech**	**Ash**	**Birch**	**Cherry**	**Fir**	**Beech**	**Ash**	**Birch**	**Cherry**	**Fir**
Precision	0.990	0.850	0.978	0.995	0.878	0.980	0.988	0.918	0.918	0.973
Recall	0.998	0.887	0.908	0.985	0.898	0.958	0.938	0.965	0.978	0.925
F1-Score	0.994	0.868	0.942	0.990	0.888	0.968	0.961	0.938	0.945	0.948
Accuracy	0.930					0.950				
	**AM-SPPResNet + XGBoost**					**AM-SPPResNet + SVM Classifier**				
	**Beech**	**Ash**	**Birch**	**Cherry**	**Fir**	**Beech**	**Ash**	**Birch**	**Cherry**	**Fir**
Precision	0.998	0.998	0.995	0.995	0.990	0.995	0.998	1.000	0.998	1.000
Recall	1.000	0.993	0.990	0.990	0.998	1.000	0.998	1.000	0.993	1.000
F1-Score	0.999	0.995	0.992	0.992	0.994	0.997	0.998	1.000	0.995	1.000
Accuracy	0.995					1.000				

**Table 4:** Prediction Matrix of AM-SPPResNet + SVM on Test Set.

	Beech	Ash	Birch	Cherry	Fir
Precision	1.00	0.99	0.98	1.00	0.99
Recall	1.00	0.98	0.99	0.99	0.99
F1-score	1.000	0.985	0.985	0.995	0.990
Accuracy	0.99				

**Table 5:** Prediction matrix of different models applied in identification of sawn timber tree species.

	**LBP (P = 8, R = 1) + SVM** **Classifier**					**GLCM (angle = [0°, 45°, 90°, 135°], d = [2,4]) + SVM Classifier**				
	**Beech**	**Ash**	**Birch**	**Cherry**	**Fir**	**Beech**	**Ash**	**Birch**	**Cherry**	**Fir**
Precision	0.990	0.965	0.720	0.825	0.925	0.898	0.973	0.783	0.888	0.975
Recall	0.888	0.923	0.895	0.775	0.905	0.878	0.960	0.853	0.823	0.988
F1-Score	0.936	0.943	0.798	0.798	0.915	0.887	0.966	0.816	0.853	0.981
Accuracy	0.880					0.903				
Average time of single image	21.3 ms					25.6 ms				
	**VGG16**					**ResNet50**				
	**Beech**	**Ash**	**Birch**	**Cherry**	**Fir**	**Beech**	**Ash**	**Birch**	**Cherry**	**Fir**
Precision	0.970	0.968	0.913	0.963	0.860	0.858	0.920	0.943	0.968	0.993
Recall	0.955	0.828	0.828	0.970	0.953	0.965	0.983	0.948	0.745	0.908
F1-Score	0.962	0.861	0.860	0.966	0.889	0.891	0.945	0.941	0.757	0.946
Accuracy	0.910					0.908				
Average time of single image	10.5 ms					35.5 ms				
	**ResNet101**					**AM-SPPResNet + SVM** **Classifier**				
	**Beech**	**Ash**	**Birch**	**Cherry**	**Fir**	**Beech**	**Ash**	**Birch**	**Cherry**	**Fir**
Precision	0.895	0.900	0.970	0.970	0.990	0.995	0.998	1.000	0.998	1.000
Recall	0.970	0.995	0.875	0.833	0.953	1.000	0.998	1.000	0.993	1.000
F1-Score	0.919	0.939	0.908	0.863	0.971	0.997	0.998	1.000	0.995	1.000
Accuracy	0.925					1.000				
Average time of single image	30.5 ms					63.8 ms				

**Table 6:** Tree species in “Wood AUTH” dataset.

Class Index	Botanical Name	Category
1	*Fagus sylvatica*	Diffuse-porous hardwood
2	*Juglans regia*	Semi-diffuse-porous hardwood
3	*Castanea sativa*	Ring-porous hardwood
4	*Quercus cerris*	Ring-porous hardwood
5	*Alnus glutinosa*	Diffuse-porous hardwood
6	*Fraxinus ornus*	Ring-porous hardwood
7	*Picea abies*	Softwood
8	*Ailanthus*	Softwood
9	*altissima*	Ring-porous hardwood
10	*Robinia pseudoacacia*	Ring-porous hardwood
11	*Cupressus sempervirens*	Softwood
12	*Platanus orientalis*	Diffuse-porous hardwood

**Table 7:** Prediction matrix of different models applied in identification of “Wood AUTH” dataset.

**VGG16**												
	**1**	**2**	**3**	**4**	**5**	**6**	**7**	**8**	**9**	**10**	**11**	**12**
Precision	0.84	0.73	0.89	0.91	0.80	0.84	0.94	0.91	0.92	0.75	0.92	0.83
Recall	0.95	0.88	0.85	0.89	0.78	0.85	0.91	0.92	0.73	0.92	0.88	0.63
F1-score	0.89	0.80	0.87	0.90	0.79	0.84	0.92	0.91	0.81	0.83	0.90	0.72
Accuracy	0.85											
Average time of single image	6.2 ms											
**ResNet50**												
	**1**	**2**	**3**	**4**	**5**	**6**	**7**	**8**	**9**	**10**	**11**	**12**
Precision	0.98	0.76	0.94	0.95	0.95	0.79	0.87	0.71	1.00	1.00	0.95	0.95
Recall	0.96	0.98	0.92	0.93	0.83	0.88	0.79	1.00	0.80	0.94	0.99	0.91
F1-score	0.97	0.86	0.93	0.94	0.89	0.83	0.83	0.83	0.89	0.97	0.97	0.93
Accuracy	0.92											
Average time of single image	9.4 ms											
**ResNet101**												
	**1**	**2**	**3**	**4**	**5**	**6**	**7**	**8**	**9**	**10**	**11**	**12**
Precision	0.94	1	0.89	0.8	0.96	0.95	0.89	0.93	1	0.97	0.98	0.89
Recall	0.91	0.92	0.96	0.97	0.81	0.79	0.98	0.98	0.86	0.95	0.96	0.95
F1-score	0.92	0.96	0.92	0.88	0.88	0.86	0.93	0.95	0.92	0.96	0.97	0.92
Accuracy	0.92											
Average time of single image	17.5 ms											
**AM-SPPResNet**												
	**1**	**2**	**3**	**4**	**5**	**6**	**7**	**8**	**9**	**10**	**11**	**12**
Precision	0.98	0.98	0.93	0.99	0.94	0.98	0.96	0.97	0.93	0.95	0.94	0.97
Recall	0.99	0.85	0.96	0.83	0.97	0.97	0.99	0.91	1	0.95	0.98	0.95
F1-score	0.98	0.91	0.94	0.90	0.95	0.97	0.97	0.94	0.96	0.95	0.96	0.96
Accuracy	0.96											
Average time of single image	15.9 ms											
**AM-SPPResNet + SVM**												
	**1**	**2**	**3**	**4**	**5**	**6**	**7**	**8**	**9**	**10**	**11**	**12**
Precision	0.98	1	0.98	1	0.98	1	0.98	1	0.99	1	0.98	0.98
Recall	1	0.92	0.99	0.98	0.99	0.97	0.99	0.99	1	0.97	1	0.97
F1-score	0.99	0.96	0.98	0.99	0.98	0.98	0.98	0.99	0.99	0.98	0.99	0.97
Accuracy	0.99											
Average time of single image	26.3 ms											

## Data Availability

The data presented in this study are available on request from the corresponding author. The data are not publicly available due to our research is still in progress.
